# The Magic Curiosity Arousing Tricks (MagicCATs) database in Italian younger and middle-aged adults: Descriptive statistics and rule-based machine learning

**DOI:** 10.3758/s13428-025-02884-z

**Published:** 2025-11-19

**Authors:** Caterina Padulo, Michela Ponticorvo, Beth Fairfield

**Affiliations:** Department of Humanistic Studies, University of Naples Federico II, Via Porta Di Massa 1, 80113 Naples, Italy

**Keywords:** Personal relevance, Epistemic emotions, Curiosity, Interest, Surprise, Rating, Association rule learning

## Abstract

Epistemic emotions, and in particular curiosity, seem to enhance memory for both the specific information that stimulates the individual’s curiosity and information presented in close temporal proximity. Most studies on memory and curiosity have adopted trivia questions to elicit curiosity. However, the amount and range of interest that trivia questions elicit are unclear, and there is no established, universal trivia item pool guaranteed to elicit comparable levels of curiosity across individuals of all ages. Thus, one substantial challenge when studying curiosity is systematically inducing it in controlled experimental settings. Recently, an innovative database called Magic Curiosity Arousing Tricks (MagicCATs) has been published. This database includes 166 short magic-trick video clips that adopt different materials and is designed to induce curiosity, surprise, and interest. Here, we aimed to validate this dataset in the Italian population by reporting the basic characteristics and the norms of the magic-trick video clips in younger and middle-aged adults. We also carried out association rule learning, a rule-based machine learning and data mining method to aid understanding of the co-occurrences between the different epistemic emotions and aid researchers in stimulus selection. Association rules underline relationships or associations between the variables in our datasets and can be used in association with descriptive statistics for stimulus selection in psychological experiments.

## Introduction

A growing body of literature suggests that epistemic emotions, including surprise for something that is learned unexpectedly, curiosity for a question that remains unanswered, and interest can have a significant impact on knowledge acquisition (Brun et al., 2008) and may be crucial to learning (Pekrun & Stephens, 2012). In particular, curiosity seems to guide information-seeking behavior and has been shown to have beneficial effects on memory across the lifespan (Fairfield et al., 2015; Galli et al., 2018; Padulo et al., 2022). Moreover, high states of curiosity also seem to enhance memory for implicitly encoded unrelated information presented in close temporal proximity (Gruber et al., 2014; Muis et al., 2018a, 2018b; Murphy et al., 2021; Padulo et al., 2022; Wade & Kidd, 2019), suggesting that high curiosity improves memory for not only the specific information that stimulates the individual’s curiosity, but for information beyond the specific target of curiosity as well.

There is no unanimous agreement on definitions of curiosity (Berlyne, 1954; Collins et al., 2004; Kidd & Hayden, 2015; Litman, 2008; Loewenstein, 1994; Oudeyer & Smith, 2016; Silvia, 2008, 2012) but most researchers agree that curiosity is determined by the information that the cognitive system expects to gain and refers to the intrinsic desire that individuals have to explore the unknown, even without an apparent reason to do so. Curiosity can be described as an intrinsic motivation to explore the environment, a need to make sense of the world (Cohen et al., 1955), and a motive to reduce negative states evoked by uncertainty, novelty, arousal, or information gaps (Berlyne, 1954; Hebb, 1955; Loewenstein, 1994). More importantly, despite the lack of agreement on the definition and exact nature of curiosity (Kidd & Hayden, 2015), studies have widely recognized a key role of curiosity in education, scientific progress, and other domains of human activity. For example, Von Stumm et al. (2011) demonstrated the importance of curiosity for scholarly achievement. Reio and Wiswell (2000) found that curiosity influences learning associated with socialization as well as technical and interpersonal dimensions of job performance. Sakaki et al. (2018), in their literature review, claim that although curiosity generally declines with age, it may be a crucial key in achieving adaptive aging.

Studies on memory and curiosity have generally adopted trivia questions to elicit curiosity (Baranes et al., 2015; Fandakova & Gruber, 2021; Halamish et al., 2019; Kang et al., 2009; Metcalfe et al., 2017; Murayama & Kuhbandner, 2011; Padulo et al., 2022; Swirsky et al., 2021). However, one limitation of the trivia question paradigm is that there is no established common trivia item pool guaranteed to induce curiosity across individuals of all ages. Furthermore, the amount and range of interest that trivia questions induce is unclear, leading to difficulties in comparing results between studies. Alternatively, a few studies have adopted visual arts (Silvia, 2005), blurred pictures (Jepma et al., 2012), philosophical quotations (Fayn et al., 2019), and feeling of knowing experiences (Brooks et al., 2021) to induce curiosity.

More recently, Ozono et al. (2021) created an innovative database called Magic Curiosity Arousing Tricks (MagicCATs), a database of 166 magic-trick video clips designed to induce curiosity, surprise and interest. The video clips present a variety of magic tricks that adopt different materials including cards, cups and balls, rings, coins, etc. and that extend in length from 8 to 155 s. The video clips consisted mainly of non-verbal information, making it easier for individuals to intuitively understand the content and trigger curiosity, surprise, and/or interest regardless of language, educational, and cultural backgrounds, since it may also be possible that demographic variables such as culture, age, and/or gender also influence experiencing curiosity, surprise, and/or interest.

In the current study, we aimed to validate this dataset in the Italian population by reporting the basic characteristics and the norms of the magic trick video clips in younger and middle-aged adults. In particular, since Ozono and coauthors (2021) found significant age effects, we recruited a sample of individuals to cover adulthood, ranging from 19 to 59 years of age. We also carried out association rule learning, a rule-based machine learning and data mining method, in the intent to identify the “rules” that determine how and why certain items of interest are connected to further the understanding of co-occurrences between curiosity, surprise and interest to further aid researchers in stimulus selection.

## Materials and methods

### Participants

A total of 550 younger and middle-aged adults participated in the study. After exclusion criteria, the final sample comprised 424 participants. Specifically, 228 younger adults (75% female) with an average age of 22.2 years (SD = 3.3) and 196 middle-aged adults (60% female) with an average age of 53.7 years (SD = 4.4) completed the rating task. All participants were native Italian speakers with normal or corrected-to-normal vision. All participants were recruited through posted advertisements among students from all departments of the university, via community outreach at events and through word of mouth. The experimental protocol was implemented in Qualtrics and distributed through an anonymous link. Participants were not paid for their participation in the study.

In line with Ozono et al. (2021), exclusion criteria for collected data included identical ratings on more than three questions for all trials, a “no” answer to the “clarity of the trick” question for all the videoclip evaluated, technical issues in video presentation and/or Internet connection, distraction during the experiment and prior participation in a similar experiment.

### Stimuli

We included all 166 videos of the original dataset created by Ozono et al. (2021) as stimuli. The videos that contained English subtitles in the original database were modified to replace the English subtitles with Italian subtitles, controlling for the correctness of the translation. As in the original database, the faces of the magicians were obscured in order to avoid potential distraction due to their appearance, facial expressions, and/or effects related to gender. All videos were then divided into nine lists, identical to those in Ozono et al. (2021).

### Procedure

Participants were randomly assigned to one of the nine lists. For each video clip, participants were asked to give five different ratings including (1) clarity, that is whether they understood the intention of the magic trick; (2) surprise, that is how surprised they were; (3) interest, that is how interesting they found the magic trick; (4) confidence, that is how confident they were about understanding the solution; and (5) curiosity, that is how curious they were about how the magic trick was done. Clarity of the trick was rated using a binary response scale (Yes/No). The other four questions were rated on ten-point Likert scales ranging from 1 (not at all) to 10 (very much). At the end of the experimental session, all participants answered three questions regarding their general interest in magic tricks, whether they had ever performed magic tricks before and eventual Internet/video problems. As done in Ozono et al. (2021), these questions were used as exclusion criteria for participants from the main data analysis.

## Results

Analyses on the post hoc questions showed that participants were generally interested in the magic tricks (M = 5.2, SD = 2.6). No participant indicated that they frequently performed magic tricks; 213 younger adults and 203 middle-aged adults indicated that they had never performed a magic trick.

Table 1 reports the descriptive statistics of the main variables (clarity, surprise, interest, confidence in the solution, and curiosity) by group for each video clip.

**Table 1:** Descriptive statistics for variables, separately for younger (G1) and middle-aged adults (G2)

	Clarity of the trick		Surprise in response to the trick		Interest in the trick		Confidence in the solution		Curiosity in the solution	
	*G1*	*G2*	*G1*	*G2*	*G1*	*G2*	*G1*	*G2*	*G1*	*G2*
Mean	.77	.69	4.82	4.42	3.71	3.21	4.81	4.51	4.75	4.4
SD	.1	.1	0.69	0.66	0.65	0.73	0.65	0.71	0.7	0.26
Min	.38	.32	3.1	2.84	2.48	1.7	3	1.52	3.13	2.89
Max	1	.94	6.6	6.1	5.67	5.24	6.17	6	6.32	6.16

On average both younger and middle-aged adults understood the intentions of the magic tricks the majority of the time (77% and 69%, respectively). Forty video clips were understood by less than 70% in the younger adults and 75% of the middle-aged adults.

Clarity differed between younger and middle-aged adults (*U* = 19,015, *Z* =  − 2.51 *p* = 0.010, with a small effect size *r* =  − 0.12). The magic tricks were clearer for younger adults (80%) than middle-aged adults (70%). Surprise differed between younger and middle-aged adults (*U* = 19,525, *Z* =  − 2.17, *p* = 0.030, with a small effect size *r* =  − 0.11), with younger adults (M = 4.8, SD 2.4) generally being more surprised than middle-aged adults (M = 4.4, SD 2.4). Interest also differed between younger and middle-aged adults (*U* = 17,758, *Z* =  − 3.58, *p* <.001, with a small effect size *r* =  − 0.174), with younger adults (M = 3.7, SD 2.4) generally showing more interest than middle-aged adults (M = 3.2, SD 2.2). Confidence in the solution was comparable between younger and middle-aged adults (M = 4.8, SD 2.7, M = 4.5, SD 2.6, respectively). Finally, curiosity showed a tendency to differ (*p* =.05) between younger and middle-aged adults since younger adults (M = 4.7, SD 2.5) tended to show more curiosity than middle-aged adults. (M = 4.4, SD 2.5). Effect sizes were small but there were substantial individual differences in which magic tricks were found surprising, curious, and interesting by participants (see Fastrich et al., 2018, for similar findings with trivia questions). Therefore, when examining epistemic emotions with these stimuli, it may be ideal to assess these epistemic emotions on a participant-by-participant basis.

As done in Ozono et al. (2021), we applied linear mixed-effects models to estimate the variance structure underlying participants’ emotional responses to video stimuli. In the initial analysis, separate models were fit for each emotion (surprise, interest, confidence, curiosity), including random intercepts for participants (SBJ) and video clips (trick). Results, shown in Table 2, revealed a substantial proportion of variance attributable to stable interindividual differences, with participant-level variance ranging from 25.7% (interest) to 36.4% (confidence). On the contrary, video-level variance was consistently small, ranging from 1.0% to 3.2%, suggesting a limited influence of stimulus-specific features on emotional responses. The largest source of variance in all models was associated with the participant × video interaction (ranging from 62.6% to 71.1%), which includes both individual-specific responses to specific stimuli and residual measurement error. These findings indicate that epistemic emotions are not uniformly evoked by specific videos but are instead shaped by idiosyncratic patterns of person–stimulus interaction.

**Table 2:** Variance components of ratings

	Participant variance	Video clip variance	Participant × video variance
Surprise	33.3%	2.5%	64.3%
Interest	25.7%	3.2%	71.1%
Confidence	36.4%	1.0%	62.6%
Curiosity	31.2%	2.2%	66.5%

To refine the decomposition of these variance components and minimize measurement error contamination, we estimated a three-way mixed-effects model (participant × video × emotion type), excluding confidence, which was not conceptualized as a core epistemic emotion (Table 3). In line with Ozono et al. (2021), we removed the random intercept for emotion type due to its negligible variance and convergence issues. The final model revealed that the largest portion of variance was accounted for by the residual component (49%), followed by the participant × video interaction (16.3%), the participant × emotion type interaction (15%), and participant-level variance (16.4%). The variance associated with video-level effects and the video × emotion type interaction was minimal (0.0% and 3.3%, respectively). Overall, results suggest that epistemic emotion responses to magic tricks are predominantly determined by individual-level factors and their interaction with specific content, rather than by intrinsic features of the stimuli themselves. These findings reinforce the importance of modeling within-person variability when studying complex emotional constructs.

**Table 3:** The variance components of each factor and their interactions

Participant	16.4%
Video	0.0%
Emotion type	-
Participant × video	16.3%
Video × emotion type	3.3%
Participant × emotion type	15.0%
Participant × video × emotion type	49.0%

To further examine the differentiation of these epistemic emotions, we also computed the correlations between surprise, interest, confidence in the solution, and curiosity (Table 4). As is typical with correlations of aggregated scores (Robinson, 1950), the between-person correlation between epistemic emotions is very high.

**Table 4:** Spearman correlations for epistemic emotions

	2	3	4
1. Surprise	0.412***	0.758***	0.886***
2. Interest		0.416***	0.365***
3. Confidence			0.818***
4. Curiosity			

* p < .05, ** p < .01, *** p < *.001*

## Association rules

In the present study, an adapted version of the balanced incomplete block (BIB) spiraling procedure (Fleiss, 1981; Hanani, 1961) was used to investigate relationships between variables. Since participants in our study did not view each of the stimuli but rather a subset of the entire database and considering the nature of the video clips, a traditional validation procedure is not suitable. Thus, together with the descriptive statistics reported above, we looked for association rules between different ratings made by participants on the proposed set of stimuli.

Specifically, we used a technique based on rule-based machine learning (RBML), a term in computer science intended to encompass any machine learning method to automatically identify regularities in data that can be expressed in the form of an IF–THEN rule (Fürnkranz et al., 2012). This is in contrast to other machine learning methods that commonly identify a singular model that can be universally applied to any instance in order to make a prediction. Depending on the type of rule found, we can discriminate between descriptive rule discovery, which aims at describing significant patterns in the given dataset in terms of rules, and predictive rule learning (Fürnkranz & Kliegr, 2015). Moreover, rule-based systems capture complex relationships between features with nonlinear relationships. They are more flexible, as they are non-parametric. In other words, they do not make strong assumptions about the underlying data distribution contrary to regression models, in which if the assumptions are violated, the model may perform poorly or lead to biased results.

Accordingly, we arranged our data to have participants’ responses aggregated for variables (clarity, surprise, interest, confidence in the solution, curiosity) and dichotomize responses. Clarity was rated using a binary response scale (Yes/No), whereas the other four questions were rated on ten-point Likert scales ranging from 1 (not at all) to 10 (very much). We dichotomized the ratings on a Likert scale by substituting higher scores (6 to 10) with Yes and lower scores (1 to 5) with NO.

We used the software Orange (Demšar et al., 2013) to find association rules. The function implements a frequent pattern mining algorithm (Han et al., 2004). To find classification rules, the algorithm generates rules for the whole itemset and skips the rules where there is no match between the consequent and one of the values.

We set the following criteria for rule induction:

- Minimal support = 70%
- Minimal confidence = 90%
- Max. number of rules = 10.000

Minimal support is the percentage of the entire data set covered by the entire rule with antecedent whereas consequent and minimal confidence is the proportion of the number of examples that fit the right side (consequent) among those that fit the left side (antecedent).

The number of rules that contained one of the cited variables is reported in Table 5.

**Table 5:** Number variables and associated rules from MagicCATs

Variable	Number of rules
Clarity of the trick (C_I)	1.879
Surprise in response to the trick (SUR)	2.086
Interest in the trick (INT)	2.071
Confidence in the solution (C_C)	2.085
Curiosity of the solution (CUR)	1.879

For the analysis of the association rules, we calculated the co-occurrence of the five cited variables. Results are reported in Table 6.

**Table 6:** Number of co-occurrences in association rules in MagicCATs variables

	C_I	SUR	INT	C_C	CUR
clarity of the trick (C_I)	430	394	432	394	435
surprise at the trick (SUR)		348	391	352	394
interest in the trick (INT)			424	391	433
confidence in the solution (C_C)				348	394
curiosity of the solution (CUR)					430

The co-occurrence table is not connected to a probability distribution but does provide interesting insights.

In sum, frequencies indicate that confidence in the solution of one trick is not very frequently associated with confidence in the solution of other tricks, indicating that there is a different level of this variable in the stimuli set. Differently, clarity, curiosity, and interest in one trick are linked to clarity, curiosity, and interest in the other tricks. Moreover, surprise is frequently associated with interest in the trick and curiosity in the solution, in line with previous studies.

See Appendix Tables 7 and 8 for means and SD of the database ratings by variable and group, respectively.

## Discussion

Here we present an Italian validation of the MagicCATs database of 166 magic-trick videos intended to elicit epistemic emotions. The videos include a variety of magic tricks that adopt different materials and that vary in length from 8 to 155 s, allowing researchers to easily choose and adopt different subsets of stimuli according to specific research goals. We provided ratings for clarity, surprise, interest, confidence in the solution and curiosity for the video clips in younger and middle-aged Italian adults. Rating results confirmed sufficient within-person variance with moderate mean levels of epistemic emotions, meaning that these video clips can be used to examine these emotions in the Italian population, especially in the younger population.

One point worth commenting on is that curiosity for magic tricks seems to be influenced by age, since middle-aged adults tended to rate videos differently compared to younger counterparts, who rated the videos as clearer, more surprising, more interesting, and more curious than middle-aged adults. This finding is contrary to the age effect observed by Ozono and colleagues, who found consistently higher overall epistemic emotion scores of older participants with respect to their younger counterparts (2021). Results from studies that directly tested the association between age and curiosity are quite mixed. Indeed, while some studies, using trivia questions, are in line with Ozono et al.’s findings (Fastrich et al., 2018; McGillivray et al., 2015), other studies found an age-related decline in curiosity (Mascherek & Zimprich, 2012) and interests (Zimprich et al., 2009).

One explanation for this may be linked to age-related declines in various brain regions. Neurological studies suggest that curiosity is related to activations in various brain regions, including the reward, dopaminergic, and noradrenergic systems, all of which are susceptible to age-related declines (see review by Sakaki et al., 2018). A complementary explanation may be linked, instead, to age-related motivational changes. According to the socioemotional selectivity theory (SST; Carstensen, 1992; Carstensen and Turk-Charles, 1994; Carstensen et al., 1999), people have two broad goal categories:

- to acquire knowledge, seek novelty, and expand breadth of knowledge, and
- to regulate negative, and maintain positive, emotional states. 

These goals operate across the lifespan so that when individuals are younger or perceive their future time as more open-ended, they are more likely to focus on information-seeking goals over emotion-regulation goals in preparation for the uncertain future. In contrast, people are more likely to favor emotion-regulation goals and psychological well-being when they perceive time as being limited. Both these explanations are consistent with the notion that curiosity declines with advanced age.

Differently, other studies suggest that curiosity may play a critical role in maintaining cognitive functioning, well-being, and physical health in older adults (e.g., Galli et al., 2018). In fact, it may also be that personal relevance changes across the lifespan and cultures. According to the selective engagement theory (Hess, 2014), age is associated with a decline in cognitive capacity, which contributes to disengagement from cognitively demanding tasks, including everyday activities. In this domain, studies show that individuals with cognitive declines exhibited less perceptual curiosity and exploratory visual behaviors than people without cognitive declines (Daffner et al., 1992). In other words, with age and age-related cognitive changes, people are likely to disengage from cognitively demanding activities and exploratory visual behaviors. Most importantly, Hess et al. (2001) identified personal relevance as one of the key moderators of the association between age and engagement since they found that older adults tended to allocate more cognitive resources in personally relevant situations and were less willing in irrelevant situations. In particular, results highlighted that older adults were more likely to engage in systematic information searches when the context was more personally relevant than when it was less personally relevant, a pattern that was not observed in younger adults.

Personal relevance can be defined as personal meaningfulness or a sort of connection between an individual and a stimulus that is personally meaningful according to personal association, personal usefulness, and identification. Personal association is a sense of connection between a stimulus and oneself and/or one’s life; personal usefulness is the perception that a stimulus may help to achieve personal goals; and identification is the incorporation of a stimulus into one’s identity.

In line with this, it may be that the middle-aged adults in our study did not consider magic tricks as personally relevant to the same degree as the younger adults. Consequently, magic tricks were simply information that taxed the cognitive system, a system already taxed by age, and therefore produced lower curiosity ratings. Indeed, magic tricks are designed to exploit cognitive biases and manipulate perception to create the illusion of magic. This illusion then directs attention away from the “secret moves” that compose the actual trick to something entirely different (Macknik et al., 2008; Quiroga, 2016). Magic tricks, then, basically create confusion during which anything may be possible, and therefore becomes irrelevant or interfering information. In sum, it could be that magic tricks may induce a lower amount of information-seeking behavior, known to lead to higher curiosity states, as age increases.

In addition, results from the descriptive association rules revealed how curiosity, interest, and clarity seem to be associated. If the illusion created by the magic trick is clear and interesting, the trick can elicit curiosity. Although middle-aged adults express, in general, more conservative ratings compared to their younger counterparts, it is possible to identify high and low state curiosity tricks in the database in the Italian population. It is important, however, to pay close attention to stimulus selection. If the magic trick is unclear, it creates confusion in a way that as age increases, curiosity in general may decline. Researchers need to consider this when selecting the tricks to adopt as stimuli.

These frequency patterns suggest that confidence in solving a trick is not often associated with confidence in solving other tricks, indicating that this variable is inconsistent across the stimulus set. However, clarity, curiosity, and interest in one trick are consistently linked to the same attributes in other tricks, suggesting that more stimuli in the dataset share these features. Furthermore, surprise is frequently associated with interest in the trick and curiosity about the solution, which aligns with findings from previous studies.

Specifically, when a magic trick creates a clear and interesting illusion, it tends to evoke curiosity. Association rules reveal relationships or associations between variables in our large dataset and can be used in association to descriptive statistics: this useful information can be used for item selection in next studies.

Finally, our study has several limitations worth discussing. One of the most important limitations of our dataset is the absence of a group of older adults (> 65 years). It would be crucial to investigate whether the pattern of performance is replicated in older adults since research shows that in some cases older adults are more curious than younger adults (Mascherek & Zimprich, 2012) and that curiosity levels may drive individual differences in crystallized intelligence (von Stumm & Deary, 2012). Also, we did not directly measure personal relevance. Future studies should consider including scales or other specific measures of self-relevance to better clarify the link between age and curiosity and the usefulness of magic tricks to induce curiosity.

## Data Availability

The data that support the findings of this study are available in the Open Science Framework. https://osf.io/j3x4s/?view_only=469429c3a1624e228dae957b96bc4da7 The final set of MagicCATs video clips with the Italian translations is available on request for research purposes.

## Appendix 1

**Table 7:** Descriptive statistics for all magic-trick videos

Magic Trick ID	No. of participants	Clarity of the trick*	Surprise in response to the trick		Interest in the trick		Confidence in the solution		Curiosity in the solution	
			Mean	SD	Mean	SD	Mean	SD	Mean	SD
K15_Long	46	0.78	4.52	2.37	4.43	2.30	3.33	2.09	5.30	2.66
K16_Short	46	0.74	5.15	2.00	3.67	2.25	5.13	2.13	4.65	2.20
K19_Long	44	0.77	5.36	2.59	3.50	2.10	5.02	2.83	5.16	2.91
K21_Short	44	0.66	3.82	2.26	4.07	2.52	4.20	2.27	3.86	2.58
K22_Long	44	0.80	4.18	2.03	4.64	2.35	4.18	2.49	4.07	2.42
K35_Short	44	0.70	4.66	2.30	3.20	2.05	4.70	2.39	4.48	2.47
S3_Long	43	0.91	4.68	2.44	4.57	2.74	4.45	2.48	4.93	2.53
S33_Short	43	0.74	5.00	2.11	3.73	2.32	5.27	2.28	5.05	2.44
H4_Short	44	0.80	4.91	2.15	3.30	2.04	5.32	2.19	5.32	2.26
Trick6_Long	44	0.70	5.20	2.41	3.07	2.30	4.73	2.69	4.93	2.64
Trick7_Short	44	0.86	5.34	2.39	3.30	2.12	5.61	2.47	5.64	2.36
Trick8_Long	43	0.91	5.16	2.50	3.36	2.25	5.34	2.57	5.61	2.70
Trick12_Short	44	0.89	5.02	2.38	4.14	2.30	4.82	2.41	4.73	2.55
Trick14_Long	43	0.65	4.91	2.47	3.23	2.16	4.82	2.52	4.86	2.39
Trick16_Short	42	0.83	5.52	2.25	3.73	2.13	5.64	2.32	5.68	2.18
Trick18_Long	44	0.86	4.30	2.34	3.55	2.45	4.57	2.26	4.57	2.62
Trick20_Short	43	0.79	5.36	2.15	3.43	2.13	5.27	2.35	5.23	2.47
Trick22_Long	44	0.86	5.05	2.48	3.36	2.06	5.30	2.57	5.09	2.67
Trick23_Long	42	0.76	5.11	2.06	3.32	2.08	5.25	2.25	4.73	2.25
Trick32_Long	44	0.61	4.48	2.60	2.73	1.91	4.91	2.73	4.91	2.60
Trick36_Short	43	0.77	5.30	2.19	3.64	2.20	5.34	2.40	5.36	2.50
K15_Short	46	0.65	4.06	2.41	3.79	2.14	2.54	2.40	4.25	2.92
K16_Long	44	0.75	4.75	2.16	3.25	2.54	4.17	2.74	4.00	2.13
K19_Short	45	0.64	4.42	2.48	2.50	2.18	4.04	2.77	4.38	2.59
K21_Long	46	0.67	3.90	2.20	3.54	2.70	4.00	2.54	4.13	2.37
K22_Short	44	0.50	3.72	2.33	2.67	2.14	3.76	2.55	4.22	2.56
K35_Long	44	0.55	3.98	2.48	3.59	2.50	3.85	2.78	3.70	2.46
S3_Short	44	0.57	3.65	2.24	2.91	2.33	3.57	2.42	3.50	2.26
S33_Long	45	0.73	3.73	2.29	3.85	2.35	3.70	2.53	3.57	2.43
H4_Long	45	0.71	4.07	2.36	2.96	2.42	3.98	2.50	4.20	2.57
Trick6_Short	45	0.62	3.91	2.26	2.70	2.20	3.76	2.64	4.02	2.49
Trick7_Long	45	0.62	4.07	2.59	2.60	2.25	3.54	2.32	3.93	2.61
Trick8_Short	45	0.69	4.76	2.66	2.91	2.24	4.57	2.83	4.57	2.62
Trick12_Long	45	0.60	3.59	2.29	2.74	2.13	3.65	2.20	3.78	2.42
Trick14_Short	44	0.45	3.87	2.47	2.56	2.06	3.47	2.38	3.76	2.54
Trick16_Long	44	0.64	3.80	2.52	2.60	2.13	3.82	2.59	3.71	2.63
Trick18_Short	43	0.58	4.00	2.45	2.52	2.16	3.59	2.50	3.93	2.48
Trick20_Long	42	0.74	4.11	2.48	2.86	2.13	4.16	2.74	3.95	2.47
Trick22_Short	42	0.81	4.77	2.50	2.72	2.07	4.12	2.72	4.28	2.75
Trick23_Short	42	0.55	3.61	2.04	2.30	1.62	3.50	2.37	3.45	2.10
Trick32_Short	43	0.79	3.61	2.29	2.75	2.30	3.25	2.53	4.00	2.69
Trick36_Long	43	0.56	3.80	2.42	2.30	1.80	3.32	2.57	3.50	2.37
K1	47	0.77	5.60	1.95	3.66	2.63	4.85	2.92	4.51	2.27
K8	47	0.74	4.57	2.17	3.77	2.39	4.47	2.62	4.45	2.24
K17	47	0.68	5.40	2.69	3.02	2.32	5.36	2.84	5.34	2.82
K29	47	0.55	4.11	2.40	3.53	2.68	4.06	2.49	3.60	2.28
S2	47	0.91	4.94	2.55	3.77	2.65	4.94	3.02	4.64	2.73
S10	46	0.76	5.48	2.32	3.74	2.70	4.93	2.63	5.26	2.31
S23	46	0.83	5.72	2.43	3.85	2.57	5.28	2.31	4.76	2.53
S25	46	0.78	4.43	2.65	3.04	2.58	4.76	2.85	4.35	2.59
S32	46	0.50	3.28	2.18	2.93	2.52	3.76	2.25	3.61	2.19
H6	46	0.83	4.91	2.44	4.02	2.70	5.04	2.54	5.22	2.59
H13	46	0.74	4.70	2.42	3.78	2.68	5.48	2.48	4.98	2.38
H20	46	0.76	4.22	2.45	4.37	2.83	4.48	2.55	4.61	2.41
H28	46	0.83	5.20	2.46	4.11	2.69	5.35	2.57	4.96	2.51
H36	46	0.76	4.96	2.77	2.91	2.43	5.13	2.86	5.33	2.78
Trick2	45	0.80	5.00	2.59	3.30	2.38	5.26	2.72	5.26	2.65
Trick13	46	0.76	4.26	2.34	3.07	2.37	3.85	2.33	4.26	2.52
Trick26	46	0.74	4.39	2.13	3.61	2.19	4.93	2.03	4.74	2.30
Trick34	45	0.62	4.78	2.40	3.16	2.13	4.58	2.64	4.69	2.73
K2	46	0.80	5.49	2.54	2.77	2.03	4.70	3.05	5.60	2.50
K9	47	0.66	3.60	2.39	4.13	2.47	3.91	2.87	3.74	2.45
K18	46	0.80	5.11	2.39	4.51	2.83	4.54	2.95	4.13	2.47
K34	46	0.65	5.34	2.72	3.06	2.03	4.38	2.99	5.32	2.48
S4	46	0.59	4.02	2.36	3.26	2.45	4.23	2.75	4.13	2.59
S11	47	0.81	5.00	2.37	3.96	2.65	4.43	2.89	4.68	2.27
S17	47	0.79	4.94	2.51	4.47	2.72	4.53	2.87	4.49	2.54
S18	46	0.54	4.43	2.45	3.33	2.40	4.65	2.89	4.37	2.68
S26	46	0.74	4.48	2.30	4.13	2.75	4.22	2.68	4.39	2.45
H7	46	0.61	4.91	2.42	3.24	2.31	4.89	2.74	4.93	2.65
H21	46	0.80	4.96	2.56	4.83	3.01	4.43	2.60	4.48	2.64
H27	45	0.73	4.57	2.71	3.22	2.32	4.57	2.83	4.93	2.69
H37	46	0.72	4.91	2.45	4.11	2.61	4.76	2.68	4.87	2.50
Trick3	46	0.72	5.24	2.61	3.33	2.53	4.78	3.03	5.17	2.73
Trick15	46	0.57	5.39	2.82	2.76	2.18	4.33	2.99	5.04	2.75
Trick27	46	0.67	4.85	2.51	3.37	2.56	4.48	2.84	5.41	2.48
Trick25	46	0.72	4.70	2.73	3.74	2.71	4.54	2.86	4.54	2.71
K10	45	0.51	3.73	2.05	3.02	2.09	4.76	2.89	3.53	2.02
K23	42	0.57	4.60	2.31	2.86	2.13	4.16	2.99	4.49	2.58
K31	42	0.83	4.93	2.15	4.77	2.52	4.40	2.39	4.26	2.20
K32	40	0.83	5.35	2.18	4.05	2.41	5.19	2.88	4.74	2.36
S5	41	0.63	5.37	2.62	4.02	2.49	5.05	3.03	5.33	2.54
S12	42	0.74	4.51	2.43	4.40	2.64	5.40	2.64	4.35	2.34
S19	42	0.76	4.07	2.51	3.86	2.52	4.74	2.84	3.91	2.58
S27	42	0.79	5.56	2.75	3.56	2.40	5.56	2.90	5.81	2.67
H8	42	0.76	4.60	2.55	3.44	2.38	5.37	2.65	4.93	2.68
H15	42	0.71	5.23	2.37	3.77	2.39	4.98	2.68	5.00	2.32
H22	42	0.79	4.95	2.22	4.19	2.54	5.33	2.78	4.72	2.14
H31	43	0.70	4.49	2.71	3.30	2.40	4.86	2.87	4.16	2.73
H38	43	0.86	4.51	2.63	3.74	2.51	4.42	2.81	4.23	2.64
Trick4	42	0.79	4.81	2.60	3.88	2.62	4.98	2.96	4.07	2.69
Trick5	43	0.67	3.81	2.27	4.05	2.76	4.33	2.71	3.74	2.34
Trick28	43	0.70	4.91	2.64	3.91	2.52	5.14	2.78	4.93	2.77
Trick37	42	0.71	5.51	2.38	3.42	2.26	5.70	2.44	5.16	2.44
K3	48	0.42	3.40	2.18	2.81	2.52	4.63	2.70	3.27	2.40
K4	49	0.78	4.53	2.38	3.49	2.51	5.29	2.71	4.76	2.63
K11	48	0.79	4.37	2.15	3.86	2.48	4.76	2.54	4.31	2.43
K24	47	0.85	4.27	2.36	4.43	2.75	4.85	2.36	4.22	2.43
S6	47	0.81	4.47	2.43	3.73	2.51	5.27	2.55	4.59	2.41
S13	49	0.80	4.57	2.31	3.31	2.29	4.84	2.55	4.67	2.38
S20	48	0.94	4.45	2.44	3.57	2.61	4.86	2.52	4.45	2.57
S28	48	0.85	4.29	2.19	3.44	2.31	4.43	2.47	4.14	2.21
H1	49	0.73	4.18	2.32	3.69	2.63	4.49	2.42	4.04	2.48
H9	49	0.73	4.88	2.61	3.92	2.72	4.58	2.52	4.63	2.67
H16	49	0.92	4.35	2.29	3.63	2.40	4.86	2.60	4.65	2.32
H24	48	0.79	4.08	2.39	3.35	2.41	4.59	2.47	4.02	2.43
H32	49	0.88	3.84	2.26	3.78	2.62	4.61	2.54	3.53	2.26
H39	48	0.77	4.92	2.33	3.42	2.31	4.73	2.58	4.60	2.51
Trick17	47	0.83	5.28	2.59	2.85	1.97	5.19	2.68	5.52	2.48
Trick19	47	0.81	4.98	2.64	3.00	2.20	4.88	2.78	4.88	2.65
Trick29	48	0.73	4.77	2.54	3.04	2.03	5.19	2.63	4.79	2.48
Trick38	47	0.87	4.74	2.35	3.70	2.43	5.04	2.37	4.85	2.39
K5	49	0.73	4.42	2.25	3.20	2.36	4.70	2.68	4.26	2.35
K12	49	0.82	5.55	2.28	4.67	2.82	5.24	2.59	4.88	2.54
K25	49	0.86	4.02	2.27	4.24	2.75	4.37	2.60	3.76	2.23
K33	49	0.65	5.53	2.59	2.45	1.66	4.63	2.92	4.90	2.61
S7	49	0.69	4.14	2.45	3.94	2.92	4.06	2.59	3.80	2.38
S14	49	0.78	3.88	2.37	4.37	2.94	4.37	2.49	3.59	2.12
S21	48	0.83	5.20	2.12	4.16	2.62	5.00	2.58	5.12	2.16
S29	49	0.69	5.22	2.59	3.20	2.23	5.00	2.75	4.92	2.73
H2	49	0.88	4.24	2.52	4.67	2.93	4.37	2.73	4.16	2.42
H10	49	0.86	4.61	2.60	4.18	2.55	4.61	2.76	4.61	2.64
H17	49	0.71	4.33	2.53	3.47	2.58	4.49	2.56	4.53	2.51
H25	49	0.73	4.92	2.52	3.24	2.33	4.67	2.73	4.47	2.56
H33	49	0.73	5.08	2.53	3.67	2.34	4.76	2.50	4.84	2.51
H40	49	0.80	5.00	3.01	2.80	2.30	5.04	2.99	4.84	2.96
Trick9	48	0.81	4.71	2.52	3.57	2.62	4.39	2.76	4.61	2.69
Trick21	49	0.80	4.73	2.64	3.35	2.39	4.67	2.85	4.65	2.46
Trick30	49	0.82	4.82	2.57	2.76	2.01	4.80	2.58	5.16	2.65
Trick39	48	0.79	4.94	2.64	2.83	2.11	4.90	2.78	4.83	2.60
K6	45	0.49	4.57	2.31	2.98	2.17	5.23	2.77	4.23	2.17
K13	43	0.63	5.34	2.46	2.98	2.06	5.27	2.65	4.91	2.60
K27	42	0.83	5.32	2.52	4.05	2.66	5.02	2.76	5.18	2.55
S8	43	0.72	5.09	2.49	3.11	2.25	5.75	2.52	5.30	2.42
S15	43	0.74	5.45	2.25	3.64	2.47	5.30	2.70	5.27	2.47
S22	43	0.81	4.93	2.64	3.98	2.56	5.09	2.73	4.86	2.66
S30	43	0.77	4.64	2.19	4.02	2.45	5.32	2.34	5.20	2.17
H3	43	0.74	4.11	2.38	4.23	2.84	4.55	2.65	4.41	2.30
H11	43	0.65	4.68	2.58	2.77	2.11	4.98	2.86	4.80	2.78
H14	43	0.77	4.68	2.40	4.70	2.63	5.41	2.41	4.59	2.39
H18	43	0.60	4.93	2.86	2.95	2.53	5.07	2.86	4.91	2.89
H26	43	0.58	4.48	2.43	3.30	2.39	4.80	2.42	4.25	2.57
H34	43	0.53	4.20	2.40	2.55	1.93	4.57	2.42	4.45	2.44
H41	40	0.78	4.79	2.40	3.44	2.23	5.16	2.61	4.91	2.58
Trick10	43	0.63	4.47	2.48	3.16	2.33	4.67	2.79	4.44	2.64
Trick24	42	0.74	4.42	2.35	4.81	2.74	4.95	2.73	4.67	2.43
Trick31	43	0.72	4.74	2.20	3.55	2.35	5.07	2.47	4.74	2.26
Trick40	42	0.64	4.86	2.80	3.26	2.60	4.90	2.99	4.71	2.74
K7	46	0.72	3.64	2.50	2.91	2.16	4.39	2.79	3.83	2.66
K14	45	0.80	4.76	2.48	3.67	2.58	4.96	2.72	5.28	2.60
K28	45	0.76	4.53	2.55	3.91	2.26	4.64	2.68	4.42	2.63
K30	45	0.80	4.96	2.84	3.82	2.41	5.02	2.77	4.93	2.79
S1	44	0.86	4.64	2.36	3.51	2.19	4.98	2.61	4.87	2.43
S9	44	0.73	4.33	2.71	3.38	2.25	4.22	2.64	4.87	2.69
S16	45	0.71	3.78	2.53	3.22	2.44	4.27	2.83	3.67	2.68
S31	44	0.82	3.42	2.20	4.07	2.64	3.69	2.48	3.58	2.37
H5	44	0.66	3.84	2.56	3.02	2.25	4.49	2.73	4.00	2.69
H12	43	0.65	4.73	2.90	2.52	1.89	4.45	3.02	4.66	2.84
H19	43	0.81	4.95	2.61	3.61	2.46	5.14	2.95	4.55	2.66
H29	44	0.80	4.68	2.39	4.02	2.25	4.70	2.62	4.82	2.70
H35	44	0.77	5.20	2.66	3.05	2.25	5.30	2.69	5.32	2.54
Trick1	43	0.63	4.48	2.56	3.02	2.20	4.25	2.53	4.82	2.58
Trick11	43	0.79	4.95	2.48	3.34	2.35	4.95	2.84	5.27	2.72
Trick25	43	0.86	4.53	2.23	3.33	2.43	4.14	2.61	4.53	2.59
Trick33	42	0.69	4.84	2.89	2.60	1.90	5.02	2.69	5.35	2.95
Trick41	43	0.47	3.37	2.57	2.63	2.06	3.51	2.75	3.53	2.80

Rating scales ranged from 1 to 10. We first computed the means of each video clip across participants and then computed the descriptive statistics. We reported the number of participants who evaluated each video clip and the mean and standard deviation for each variable

**Table 8:** Descriptive statistics separate for younger and middle-aged adults, for all magic trick videos

Magic Trick ID	No. of participants		Clarity of the trick*		Surprise in response to the trick				Interest in the trick	
	G1	G2	G1	G2	G1		G2		G1	
					Mean	SD	Mean	SD	Mean	SD
K15_Long	26	20	0.77	0.80	4.58	2.28	4.45	2.54	4.77	2.18
K16_Short	26	20	0.62	0.90	5.31	1.78	4.95	2.28	3.58	2.28
K19_Long	25	19	0.80	0.74	5.20	2.81	5.58	2.32	3.68	2.27
K21_Short	25	19	0.64	0.68	3.56	2.10	4.16	2.48	4.00	2.72
K22_Long	25	19	0.76	0.84	3.96	2.07	4.47	1.98	4.48	2.33
K35_Short	25	19	0.68	0.74	4.24	2.03	5.21	2.57	3.52	2.16
S3_Long	25	18	0.96	0.83	4.92	2.47	4.37	2.43	4.56	2.83
S33_Short	25	18	0.68	0.83	4.52	2.08	5.63	2.03	4.00	2.60
H4_Short	25	19	0.76	0.84	5.08	2.29	4.68	2.00	3.12	2.07
Trick6_Long	25	19	0.64	0.79	5.36	2.33	5.00	2.56	3.08	2.33
Trick7_Short	25	19	0.88	0.84	5.60	2.65	5.00	2.03	3.48	2.28
Trick8_Long	25	18	0.88	0.94	4.96	2.76	5.42	2.14	3.40	2.50
Trick12_Short	25	19	0.96	0.79	5.20	2.45	4.79	2.32	4.16	2.48
Trick14_Long	25	18	0.60	0.72	4.96	2.57	4.84	2.39	3.40	2.22
Trick16_Short	25	17	0.88	0.76	5.60	2.43	5.42	2.04	4.24	2.24
Trick18_Long	25	19	0.84	0.89	4.44	2.40	4.11	2.31	3.72	2.54
Trick20_Short	25	18	0.76	0.83	5.16	2.19	5.63	2.11	3.64	2.25
Trick22_Long	25	19	0.84	0.89	4.96	2.61	5.16	2.36	3.48	2.35
Trick23_Long	25	17	0.72	0.82	5.04	2.26	5.21	1.81	3.52	2.37
Trick32_Long	25	19	0.52	0.74	4.68	2.63	4.21	2.62	2.92	1.82
Trick36_Short	25	18	0.76	0.78	5.16	2.12	5.47	2.34	4.28	2.34
K15_Short	26	20	0.81	0.45	4.59	2.50	3.38	2.16	4.26	2.28
K16_Long	27	17	0.81	0.65	4.85	1.79	4.62	2.60	3.63	2.73
K19_Short	27	18	0.70	0.56	4.85	2.36	3.86	2.57	3.00	2.51
K21_Long	27	19	0.78	0.53	4.70	2.20	2.86	1.74	4.19	2.80
K22_Short	25	19	0.60	0.37	3.92	2.29	3.48	2.40	3.00	2.29
K35_Long	25	19	0.60	0.47	4.32	2.38	3.57	2.60	4.28	2.78
S3_Short	25	19	0.68	0.42	3.40	2.08	3.95	2.44	3.24	2.60
S33_Long	25	20	0.76	0.70	3.56	2.20	3.95	2.44	4.08	2.50
H4_Long	25	20	0.80	0.60	4.72	2.25	3.29	2.31	3.40	2.77
Trick6_Short	25	20	0.72	0.50	4.36	2.18	3.38	2.29	3.04	2.34
Trick7_Long	25	20	0.76	0.45	4.64	2.25	3.38	2.84	3.17	2.43
Trick8_Short	25	20	0.76	0.60	5.32	2.44	4.10	2.81	3.40	2.29
Trick12_Long	25	20	0.72	0.45	4.20	2.25	2.86	2.15	3.24	2.24
Trick14_Short	25	19	0.56	0.32	3.84	2.34	3.90	2.69	3.24	2.44
Trick16_Long	25	19	0.68	0.58	3.64	2.51	4.00	2.58	3.12	2.60
Trick18_Short	25	18	0.64	0.50	4.28	2.48	3.63	2.43	2.60	2.16
Trick20_Long	24	18	0.75	0.72	4.20	2.22	4.00	2.85	3.24	2.30
Trick22_Short	24	18	0.88	0.72	4.67	2.22	4.89	2.87	3.38	2.24
Trick23_Short	25	17	0.68	0.35	4.20	2.06	2.84	1.77	2.48	1.61
Trick32_Short	25	18	0.88	0.67	3.68	2.30	3.53	2.34	2.64	2.40
Trick36_Long	25	18	0.60	0.50	3.84	2.30	3.74	2.62	2.72	2.17
K1	22	25	0.77	0.76	5.64	2.08	5.56	1.87	3.86	2.75
K8	22	25	0.82	0.68	4.95	2.10	4.24	2.22	4.23	2.62
K17	22	25	0.68	0.68	6.32	2.66	4.60	2.50	2.77	2.02
K29	22	25	0.59	0.52	4.36	2.42	3.88	2.40	3.55	2.89
S2	22	25	0.95	0.88	5.23	2.67	4.68	2.46	3.32	2.44
S10	21	25	0.90	0.64	6.00	2.17	5.04	2.39	3.90	2.77
S23	21	25	0.86	0.80	6.00	2.12	5.48	2.68	4.57	2.44
S25	21	25	0.76	0.80	5.00	2.59	3.96	2.65	3.57	2.77
S32	21	25	0.43	0.56	3.10	1.97	3.44	2.36	3.10	2.55
H6	21	25	0.90	0.76	4.86	2.48	4.96	2.46	4.95	2.46
H13	21	25	0.67	0.80	5.05	2.50	4.40	2.36	3.33	2.15
H20	21	25	0.81	0.72	4.67	2.42	3.84	2.46	4.81	2.64
H28	21	25	0.95	0.72	5.95	2.46	4.56	2.31	5.67	2.54
H36	21	25	0.81	0.72	5.33	2.63	4.64	2.90	3.57	2.73
Trick2	21	24	0.86	0.75	5.67	2.71	4.44	2.40	3.52	2.06
Trick13	21	25	0.81	0.72	4.10	2.28	4.40	2.43	3.48	2.38
Trick26	21	25	0.81	0.68	4.14	2.26	4.60	2.04	3.90	2.28
Trick34	20	25	0.60	0.64	5.11	2.49	4.52	2.35	3.74	2.16
K2	25	21	0.92	0.67	6.00	2.21	4.86	2.83	2.88	1.95
K9	26	21	0.65	0.67	4.12	2.45	2.95	2.20	3.96	2.66
K18	26	20	0.85	0.75	5.27	2.38	4.90	2.45	4.88	2.93
K34	26	20	0.69	0.60	5.46	2.98	5.19	2.42	3.38	2.04
S4	26	20	0.69	0.45	4.38	2.28	3.57	2.44	3.54	2.45
S11	26	21	0.92	0.67	5.38	2.47	4.52	2.20	3.88	2.70
S17	26	21	0.92	0.62	5.31	2.13	4.48	2.89	5.19	2.70
S18	25	21	0.44	0.67	4.48	2.35	4.38	2.62	3.08	2.38
S26	25	21	0.92	0.52	4.96	2.24	3.90	2.28	4.64	2.90
H7	25	21	0.72	0.48	5.68	2.04	4.00	2.57	3.88	2.26
H21	25	21	0.80	0.81	5.16	2.67	4.71	2.47	4.56	3.06
H27	25	20	0.72	0.75	5.28	2.65	3.71	2.57	3.44	2.29
H37	25	21	0.76	0.67	5.44	2.29	4.29	2.53	4.44	2.55
Trick3	25	21	0.76	0.67	6.08	2.22	4.24	2.74	3.84	2.73
Trick15	25	21	0.76	0.33	6.60	2.31	3.95	2.75	3.32	2.38
Trick27	25	21	0.76	0.57	5.92	2.00	3.57	2.50	3.72	2.56
Trick25	25	21	0.76	0.67	5.88	2.42	3.29	2.43	4.44	2.81
K10	23	22	0.52	0.50	3.48	2.13	4.00	1.98	3.09	2.11
K23	21	21	0.76	0.38	4.95	2.08	4.24	2.53	2.77	2.25
K31	21	21	0.90	0.76	4.77	2.22	5.10	2.12	4.32	2.53
K32	20	20	0.90	0.75	5.18	2.13	5.52	2.27	4.68	2.44
S5	21	20	0.67	0.60	5.64	2.57	5.10	2.70	3.77	2.51
S12	21	21	0.86	0.62	4.50	2.20	4.52	2.71	4.59	2.70
S19	21	21	0.86	0.67	4.05	2.82	4.10	2.21	3.95	2.48
S27	21	21	0.81	0.76	5.09	3.07	6.05	2.33	3.32	2.40
H8	21	21	0.81	0.71	4.64	2.84	4.57	2.27	3.27	2.57
H15	22	20	0.73	0.70	5.86	2.38	4.57	2.23	3.55	2.20
H22	21	21	0.81	0.76	4.05	2.34	5.90	1.67	4.55	2.76
H31	22	21	0.77	0.62	4.68	2.78	4.29	2.69	3.23	2.41
H38	22	21	0.86	0.86	4.23	2.51	4.81	2.79	3.55	2.54
Trick4	21	21	0.90	0.67	4.64	2.74	5.00	2.51	4.09	2.65
Trick5	22	21	0.73	0.62	3.82	2.24	3.81	2.36	5.18	2.97
Trick28	22	21	0.77	0.62	4.55	2.65	5.29	2.63	4.14	2.61
Trick37	21	21	0.81	0.62	5.55	2.22	5.48	2.60	3.32	2.34
K3	32	16	0.38	0.50	3.23	2.03	3.75	2.49	2.55	2.17
K4	32	17	0.81	0.71	4.38	2.42	4.82	2.35	3.66	2.56
K11	32	16	0.78	0.81	4.31	2.24	4.47	2.03	3.56	2.44
K24	32	15	0.84	0.87	4.09	2.23	4.59	2.62	4.50	2.83
S6	32	15	0.81	0.80	4.31	2.44	4.76	2.46	3.88	2.64
S13	32	17	0.78	0.82	4.75	2.38	4.24	2.19	3.31	2.43
S20	32	16	1.00	0.81	4.66	2.47	4.06	2.41	3.75	2.69
S28	32	16	0.81	0.94	4.00	2.06	4.82	2.38	3.22	2.24
H1	32	17	0.81	0.59	4.38	2.25	3.82	2.48	3.69	2.66
H9	32	17	0.72	0.76	4.94	2.73	4.76	2.46	3.94	2.69
H16	32	17	0.91	0.94	4.19	2.43	4.65	2.03	3.28	2.41
H24	32	16	0.78	0.81	4.03	2.31	4.18	2.60	3.53	2.60
H32	32	17	0.91	0.82	3.69	2.28	4.12	2.26	3.94	2.82
H39	32	16	0.78	0.75	4.91	2.41	4.94	2.24	3.63	2.49
Trick17	32	15	0.94	0.60	5.75	2.59	4.27	2.34	2.84	2.00
Trick19	32	15	0.84	0.73	4.78	2.71	5.38	2.53	2.94	2.24
Trick29	32	16	0.78	0.63	4.75	2.57	4.81	2.56	3.06	2.06
Trick38	31	16	0.94	0.75	4.55	2.42	5.13	2.25	4.00	2.54
K5	27	22	0.67	0.82	4.44	2.15	4.39	2.41	3.26	2.33
K12	26	23	0.81	0.83	5.77	1.97	5.30	2.62	5.58	2.73
K25	26	23	0.85	0.87	4.35	2.33	3.65	2.19	4.15	2.84
K33	26	23	0.62	0.70	5.54	2.44	5.52	2.81	2.54	1.61
S7	26	23	0.73	0.65	4.35	2.43	3.91	2.50	4.12	2.93
S14	26	23	0.77	0.78	3.81	2.38	3.96	2.40	4.42	3.19
S21	25	23	0.84	0.83	5.35	1.98	5.04	2.31	4.58	2.55
S29	26	23	0.73	0.65	5.96	2.36	4.39	2.64	3.54	2.23
H2	26	23	0.88	0.87	4.77	2.79	3.65	2.08	4.88	2.78
H10	26	23	0.81	0.91	5.00	2.86	4.17	2.25	4.27	2.59
H17	26	23	0.69	0.74	4.58	2.52	4.04	2.57	3.35	2.48
H25	26	23	0.69	0.78	4.77	2.52	5.09	2.57	3.42	2.30
H33	26	23	0.77	0.70	5.62	2.52	4.48	2.47	4.27	2.38
H40	26	23	0.85	0.74	5.12	3.14	4.87	2.93	2.92	2.38
Trick9	26	22	0.81	0.82	4.62	2.67	4.83	2.41	4.23	2.86
Trick21	26	23	0.81	0.78	5.23	2.80	4.17	2.37	3.42	2.45
Trick30	26	23	0.81	0.83	4.92	2.74	4.70	2.42	2.50	1.39
Trick39	25	23	0.84	0.74	4.68	2.84	5.22	2.43	2.48	1.66
K6	19	26	0.58	0.42	5.37	2.03	3.96	2.35	2.79	2.15
K13	18	25	0.67	0.60	5.89	2.11	4.96	2.65	2.83	1.92
K27	18	24	0.89	0.79	5.89	2.32	4.92	2.62	3.11	2.27
S8	18	25	0.72	0.72	4.89	2.49	5.23	2.53	3.33	2.47
S15	18	25	0.78	0.72	5.89	2.03	5.15	2.38	4.17	2.64
S22	18	25	0.94	0.72	5.33	2.33	4.65	2.84	4.06	2.41
S30	18	25	0.67	0.84	5.06	2.26	4.35	2.13	3.39	2.40
H3	18	25	0.89	0.64	4.00	2.30	4.19	2.48	4.17	2.96
H11	18	25	0.67	0.64	4.89	2.32	4.54	2.77	2.78	2.07
H14	18	25	0.89	0.68	4.72	2.52	4.65	2.37	4.83	2.46
H18	18	25	0.72	0.52	5.22	2.73	4.73	2.97	3.33	2.81
H26	18	25	0.83	0.40	5.50	2.09	3.77	2.42	4.11	2.37
H34	18	25	0.61	0.48	4.28	1.96	4.15	2.69	2.72	2.08
H41	15	25	0.80	0.76	5.47	2.12	4.35	2.50	3.76	2.49
Trick10	17	26	0.71	0.58	4.82	2.40	4.23	2.55	3.47	2.50
Trick24	17	25	0.82	0.68	4.76	2.25	4.19	2.43	5.00	2.78
Trick31	17	26	0.71	0.73	5.65	2.00	4.15	2.17	3.59	2.76
Trick40	16	26	0.63	0.65	6.25	2.11	4.00	2.86	4.56	2.94
K7	26	20	0.81	0.60	3.76	2.47	3.50	2.59	3.46	2.42
K14	26	19	0.81	0.79	5.46	2.50	3.85	2.18	3.92	2.58
K28	26	19	0.85	0.63	5.08	2.78	3.79	2.04	4.38	2.28
K30	26	19	0.81	0.79	5.00	2.87	4.89	2.88	4.46	2.21
S1	26	18	0.92	0.78	4.54	2.55	4.79	2.12	3.81	1.98
S9	25	19	0.84	0.58	4.42	2.90	4.21	2.51	3.96	2.32
S16	26	19	0.73	0.68	3.73	2.79	3.84	2.19	3.38	2.62
S31	25	19	0.88	0.74	3.69	2.41	3.05	1.87	4.12	2.52
H5	25	19	0.68	0.63	3.92	2.67	3.74	2.47	3.46	2.34
H12	24	19	0.71	0.58	4.68	3.06	4.79	2.74	3.00	1.89
H19	24	19	0.83	0.79	4.84	2.66	5.11	2.62	4.68	2.54
H29	25	19	0.76	0.84	5.04	2.47	4.21	2.25	4.20	2.10
H35	25	19	0.80	0.74	5.60	2.75	4.68	2.52	3.52	2.31
Trick1	25	18	0.64	0.61	4.68	2.48	4.21	2.70	3.60	2.24
Trick11	25	18	0.84	0.72	5.16	2.56	4.68	2.40	4.24	2.47
Trick25	24	19	0.88	0.84	4.92	2.36	4.05	2.01	3.88	2.71
Trick33	24	18	0.71	0.67	5.29	2.94	4.26	2.79	3.25	2.11
Trick41	24	19	0.46	0.47	3.54	2.60	3.16	2.59	2.96	2.14

Magic Trick ID	Interest in the trick		Confidence in the solution				Curiosity in the solution			
	G2		G1		G2		G1		G2	
	Mean	SD	Mean	SD	Mean	SD	Mean	SD	Mean	SD
K15_Long	4.00	2.43	3.38	2.19	3.25	2.00	5.62	2.80	4.90	2.47
K16_Short	3.80	2.26	5.27	2.05	4.95	2.26	4.81	2.28	4.45	2.14
K19_Long	3.26	1.88	4.80	3.11	5.32	2.47	5.36	3.17	4.89	2.58
K21_Short	4.16	2.29	3.60	2.00	5.00	2.40	3.60	2.60	4.21	2.57
K22_Long	4.84	2.43	3.56	2.53	5.00	2.24	3.24	2.17	5.16	2.36
K35_Short	2.79	1.87	4.12	2.09	5.47	2.59	3.76	2.22	5.42	2.52
S3_Long	4.58	2.69	4.28	2.53	4.68	2.47	5.20	2.65	4.58	2.39
S33_Short	3.37	1.89	5.00	2.18	5.63	2.41	4.72	2.44	5.47	2.44
H4_Short	3.53	2.04	5.44	2.29	5.16	2.09	5.60	2.38	4.95	2.09
Trick6_Long	3.05	2.32	4.44	2.65	5.11	2.77	5.16	2.69	4.63	2.63
Trick7_Short	3.05	1.93	5.36	2.50	5.95	2.46	5.28	2.70	6.11	1.79
Trick8_Long	3.32	1.95	5.60	2.58	5.00	2.58	5.60	2.89	5.63	2.50
Trick12_Short	4.11	2.11	4.76	2.35	4.89	2.56	4.72	2.54	4.74	2.62
Trick14_Long	3.00	2.11	4.96	2.57	4.63	2.50	4.96	2.48	4.74	2.33
Trick16_Short	3.05	1.81	5.40	2.63	5.95	1.87	5.32	2.34	6.16	1.89
Trick18_Long	3.32	2.38	4.72	2.17	4.37	2.41	4.60	2.75	4.53	2.50
Trick20_Short	3.16	1.98	5.16	2.30	5.42	2.46	4.84	2.51	5.74	2.38
Trick22_Long	3.21	1.65	5.64	2.56	4.84	2.57	5.12	2.80	5.05	2.55
Trick23_Long	3.05	1.65	5.56	2.18	4.84	2.34	4.56	2.50	4.95	1.90
Trick32_Long	2.47	2.04	5.12	2.64	4.63	2.89	4.56	2.68	5.37	2.50
Trick36_Short	2.79	1.72	5.56	2.22	5.05	2.66	5.44	2.50	5.26	2.56
K15_Short	3.19	1.83	3.33	2.80	1.52	1.17	4.37	2.95	4.10	2.95
K16_Long	2.76	2.23	4.56	2.81	3.67	2.63	4.04	1.97	3.95	2.38
K19_Short	1.86	1.49	4.41	2.68	3.57	2.87	4.70	2.54	3.95	2.65
K21_Long	2.71	2.37	4.26	2.58	3.67	2.52	4.33	2.25	3.86	2.54
K22_Short	2.29	1.93	4.08	2.69	3.38	2.40	4.20	2.55	4.24	2.64
K35_Long	2.76	1.87	4.44	2.92	3.14	2.50	4.08	2.50	3.24	2.39
S3_Short	2.52	1.94	3.32	2.25	3.86	2.63	3.36	2.18	3.67	2.39
S33_Long	3.57	2.18	3.56	2.35	3.86	2.78	3.24	2.15	3.95	2.73
H4_Long	2.43	1.86	4.44	2.33	3.43	2.64	4.80	2.42	3.48	2.62
Trick6_Short	2.29	2.00	3.96	2.56	3.52	2.79	4.16	2.27	3.86	2.78
Trick7_Long	1.95	1.88	3.96	2.21	3.05	2.40	4.72	2.34	3.00	2.66
Trick8_Short	2.33	2.08	4.96	2.81	4.10	2.84	4.92	2.41	4.14	2.85
Trick12_Long	2.14	1.88	4.12	2.20	3.10	2.12	4.24	2.39	3.24	2.41
Trick14_Short	1.70	0.98	3.92	2.34	2.90	2.36	3.60	2.38	3.95	2.78
Trick16_Long	1.95	1.05	3.72	2.62	3.95	2.61	3.60	2.68	3.85	2.64
Trick18_Short	2.42	2.22	3.96	2.39	3.11	2.62	4.36	2.55	3.37	2.34
Trick20_Long	2.37	1.83	4.52	2.58	3.68	2.93	3.88	2.15	4.05	2.90
Trick22_Short	1.89	1.52	4.17	2.57	4.05	2.97	4.54	2.73	3.95	2.82
Trick23_Short	2.05	1.65	4.08	2.41	2.74	2.13	3.84	2.06	2.95	2.09
Trick32_Short	2.89	2.23	3.00	2.31	3.58	2.83	3.72	2.39	4.37	3.08
Trick36_Long	1.74	0.93	3.40	2.40	3.21	2.84	3.96	2.46	2.89	2.16
K1	3.48	2.57	5.00	3.15	4.72	2.76	4.64	2.38	4.40	2.22
K8	3.36	2.14	4.73	2.66	4.24	2.62	5.05	2.24	3.92	2.16
K17	3.24	2.57	5.50	2.89	5.24	2.85	6.32	2.66	4.48	2.73
K29	3.52	2.54	4.59	2.72	3.60	2.22	3.82	2.20	3.40	2.38
S2	4.16	2.81	4.91	3.01	4.96	3.09	4.77	2.84	4.52	2.68
S10	3.60	2.68	4.86	2.48	5.00	2.80	5.62	2.25	4.96	2.37
S23	3.24	2.57	5.05	2.09	5.48	2.50	5.00	2.21	4.56	2.80
S25	2.60	2.38	5.14	2.92	4.44	2.81	5.00	2.19	3.80	2.81
S32	2.80	2.55	3.43	2.38	4.04	2.15	3.33	1.93	3.84	2.39
H6	3.24	2.68	5.00	2.72	5.08	2.43	5.29	2.59	5.16	2.64
H13	4.16	3.05	5.86	2.41	5.16	2.54	5.52	2.29	4.52	2.40
H20	4.00	2.99	5.00	2.39	4.04	2.64	5.00	2.41	4.28	2.41
H28	2.80	2.06	5.67	2.52	5.08	2.63	5.38	2.56	4.60	2.47
H36	2.36	2.04	5.90	2.59	4.48	2.96	5.81	2.79	4.92	2.76
Trick2	3.12	2.65	5.71	2.69	4.88	2.74	5.71	2.83	4.88	2.49
Trick13	2.71	2.35	4.10	2.28	3.64	2.40	4.43	2.62	4.12	2.47
Trick26	3.36	2.12	5.10	2.12	4.80	1.98	4.48	2.54	4.96	2.11
Trick34	2.71	2.03	4.85	2.62	4.36	2.69	5.60	2.98	3.96	2.32
K2	2.62	2.18	4.16	3.17	5.33	2.83	6.04	2.18	5.05	2.80
K9	4.33	2.27	3.96	2.92	3.86	2.87	4.04	2.47	3.38	2.44
K18	4.05	2.69	4.81	3.09	4.20	2.80	3.88	2.44	4.43	2.54
K34	2.67	1.98	4.08	3.11	4.76	2.86	5.73	2.44	4.81	2.48
S4	2.90	2.47	3.92	2.81	4.62	2.69	4.23	2.49	4.00	2.77
S11	4.05	2.64	4.04	3.12	4.90	2.59	4.88	2.45	4.43	2.04
S17	3.57	2.52	4.35	2.73	4.76	3.10	4.77	2.18	4.14	2.95
S18	3.62	2.46	4.84	3.13	4.43	2.64	4.88	2.82	3.76	2.43
S26	3.52	2.50	4.72	2.79	3.62	2.48	4.96	2.35	3.71	2.45
H7	2.48	2.18	5.12	2.76	4.62	2.77	5.52	2.31	4.24	2.91
H21	5.14	3.00	4.20	2.57	4.71	2.67	4.60	2.42	4.33	2.94
H27	2.95	2.38	5.04	2.99	4.00	2.59	5.48	2.60	4.29	2.70
H37	3.71	2.69	4.48	2.71	5.10	2.66	5.40	2.24	4.24	2.70
Trick3	2.71	2.17	4.80	3.07	4.76	3.06	6.04	2.26	4.14	2.94
Trick15	2.10	1.76	4.56	3.04	4.05	2.97	6.24	2.33	3.62	2.56
Trick27	2.95	2.56	4.96	3.14	3.90	2.39	6.32	2.01	4.33	2.59
Trick25	2.90	2.39	5.08	2.90	3.90	2.74	5.04	2.49	3.95	2.89
K10	2.95	2.13	4.22	2.98	5.32	2.75	3.17	1.90	3.91	2.11
K23	2.95	2.06	3.73	2.93	4.62	3.06	4.55	2.61	4.43	2.60
K31	5.24	2.47	4.32	2.57	4.48	2.25	4.36	2.17	4.14	2.29
K32	3.38	2.25	5.18	3.11	5.19	2.69	4.55	2.40	4.95	2.36
S5	4.29	2.51	4.91	3.32	5.19	2.77	5.36	2.63	5.29	2.51
S12	4.19	2.62	5.27	2.81	5.52	2.50	4.09	2.31	4.62	2.40
S19	3.76	2.62	4.32	3.11	5.19	2.52	3.77	2.76	4.05	2.44
S27	3.81	2.44	5.36	3.06	5.76	2.77	5.59	2.87	6.05	2.50
H8	3.62	2.22	5.45	2.94	5.29	2.39	5.18	2.89	4.67	2.48
H15	4.00	2.61	4.77	2.84	5.19	2.54	4.91	2.33	5.10	2.36
H22	3.81	2.29	4.73	3.12	5.95	2.27	4.00	2.14	5.48	1.91
H31	3.38	2.44	5.27	3.06	4.43	2.66	4.27	2.85	4.05	2.67
H38	3.95	2.52	4.32	2.95	4.52	2.73	4.09	2.74	4.38	2.58
Trick4	3.67	2.63	4.86	3.11	5.10	2.88	4.14	2.47	4.00	2.95
Trick5	2.86	1.96	4.59	2.82	4.05	2.62	3.36	2.15	4.14	2.52
Trick28	3.67	2.48	5.36	2.87	4.90	2.74	4.82	2.87	5.05	2.73
Trick37	3.52	2.23	5.41	2.61	6.00	2.26	4.68	2.28	5.67	2.56
K3	3.31	3.09	4.52	2.51	4.82	3.09	3.13	2.11	3.53	2.92
K4	3.18	2.46	5.34	2.74	5.18	2.72	4.50	2.66	5.24	2.56
K11	4.41	2.55	5.13	2.49	4.06	2.56	4.31	2.58	4.29	2.20
K24	4.29	2.69	4.84	2.29	4.88	2.58	4.22	2.32	4.24	2.70
S6	3.47	2.32	5.59	2.43	4.65	2.71	4.34	2.50	5.06	2.22
S13	3.29	2.08	5.00	2.57	4.53	2.58	4.69	2.47	4.65	2.29
S20	3.24	2.49	4.91	2.54	4.76	2.56	4.41	2.59	4.53	2.62
S28	3.88	2.47	4.25	2.48	4.76	2.51	4.06	2.20	4.29	2.28
H1	3.71	2.66	4.59	2.43	4.29	2.47	4.28	2.56	3.59	2.35
H9	3.88	2.85	4.35	2.50	5.00	2.57	4.39	2.72	5.06	2.61
H16	4.29	2.28	4.84	2.74	4.88	2.39	4.41	2.54	5.12	1.83
H24	3.00	2.03	4.84	2.57	4.12	2.26	4.13	2.45	3.82	2.46
H32	3.47	2.24	4.56	2.58	4.71	2.54	3.44	2.31	3.71	2.20
H39	3.00	1.93	4.81	2.55	4.56	2.73	4.53	2.60	4.75	2.38
Trick17	2.88	1.96	5.44	2.69	4.69	2.68	5.88	2.51	4.81	2.32
Trick19	3.13	2.17	5.06	2.75	4.50	2.90	4.91	2.68	4.81	2.69
Trick29	3.00	2.03	5.31	2.68	4.94	2.59	4.75	2.62	4.87	2.23
Trick38	3.13	2.16	5.00	2.49	5.13	2.19	4.68	2.56	5.19	2.04
K5	3.13	2.44	5.15	2.58	4.17	2.76	4.48	2.14	4.00	2.59
K12	3.65	2.62	5.27	2.57	5.22	2.66	4.85	2.52	4.91	2.61
K25	4.35	2.71	4.62	2.68	4.09	2.54	3.96	2.25	3.52	2.23
K33	2.35	1.75	4.38	3.01	4.91	2.86	5.23	2.44	4.52	2.79
S7	3.74	2.96	4.19	2.53	3.91	2.70	4.00	2.23	3.57	2.57
S14	4.30	2.70	4.35	2.56	4.39	2.46	3.35	2.08	3.87	2.18
S21	3.70	2.69	4.92	2.62	5.09	2.59	5.42	2.04	4.78	2.28
S29	2.83	2.21	5.73	2.82	4.17	2.48	5.88	2.52	3.83	2.59
H2	4.43	3.13	4.38	2.84	4.35	2.66	4.50	2.57	3.78	2.24
H10	4.09	2.56	4.50	2.89	4.74	2.67	4.96	2.84	4.22	2.39
H17	3.61	2.74	4.85	2.46	4.09	2.66	5.04	2.39	3.96	2.57
H25	3.04	2.40	4.35	2.73	5.04	2.74	4.62	2.47	4.30	2.70
H33	2.95	2.13	4.96	2.51	4.52	2.54	5.15	2.49	4.48	2.54
H40	2.65	2.25	4.88	3.24	5.22	2.73	4.54	3.10	5.17	2.82
Trick9	2.83	2.15	4.69	2.95	4.04	2.55	4.88	2.82	4.30	2.57
Trick21	3.26	2.36	5.00	3.07	4.30	2.58	5.31	2.48	3.91	2.27
Trick30	3.04	2.53	4.69	2.71	4.91	2.48	5.81	2.65	4.43	2.50
Trick39	3.22	2.49	4.76	2.98	5.04	2.62	4.72	2.69	4.96	2.55
K6	3.12	2.22	6.05	3.14	4.60	2.33	4.74	2.10	3.83	2.18
K13	3.08	2.19	5.50	2.71	5.12	2.66	5.22	2.18	4.69	2.88
K27	4.69	2.75	5.39	2.85	4.77	2.72	5.78	2.39	4.77	2.61
S8	2.96	2.13	6.17	2.53	5.46	2.52	5.33	2.43	5.27	2.46
S15	3.27	2.32	5.72	2.63	5.00	2.76	5.50	2.18	5.12	2.69
S22	3.92	2.70	5.39	2.68	4.88	2.80	5.50	2.48	4.42	2.74
S30	4.46	2.44	6.00	2.22	4.85	2.34	5.50	2.23	5.00	2.15
H3	4.27	2.81	5.11	2.37	4.15	2.80	4.39	2.20	4.42	2.40
H11	2.77	2.18	5.44	2.57	4.65	3.05	5.50	2.43	4.31	2.94
H14	4.62	2.79	5.61	2.52	5.27	2.38	4.67	2.59	4.54	2.28
H18	2.69	2.35	4.94	2.82	5.15	2.94	4.83	2.77	4.96	3.03
H26	2.73	2.27	6.11	1.81	3.88	2.39	4.94	2.53	3.77	2.53
H34	2.42	1.86	4.89	2.30	4.35	2.51	4.72	2.27	4.27	2.59
H41	3.23	2.07	5.94	2.54	4.65	2.58	5.24	2.59	4.69	2.60
Trick10	2.96	2.24	5.71	2.59	4.00	2.76	4.88	2.78	4.15	2.56
Trick24	4.69	2.75	5.65	2.74	4.50	2.67	5.12	2.57	4.38	2.33
Trick31	3.52	2.08	5.94	1.92	4.50	2.66	5.41	2.03	4.31	2.33
Trick40	2.46	2.02	6.06	2.46	4.19	3.11	5.94	2.38	3.96	2.72
K7	2.20	1.54	5.12	2.79	3.45	2.56	4.04	2.65	3.55	2.72
K14	3.35	2.62	5.27	2.74	4.55	2.70	5.46	2.58	5.05	2.68
K28	3.26	2.13	5.12	2.78	4.00	2.47	4.92	2.74	3.74	2.38
K30	2.95	2.46	5.50	2.80	4.37	2.65	5.04	2.88	4.79	2.74
S1	3.11	2.45	4.96	2.65	5.00	2.62	4.85	2.69	4.89	2.08
S9	2.58	1.92	4.12	2.60	4.37	2.75	5.23	2.70	4.37	2.67
S16	3.00	2.21	4.77	3.05	3.58	2.41	3.85	2.81	3.42	2.55
S31	4.00	2.87	4.15	2.63	3.05	2.15	4.08	2.45	2.89	2.13
H5	2.42	2.04	4.77	2.80	4.11	2.64	4.42	2.76	3.42	2.55
H12	1.89	1.73	4.88	3.18	3.89	2.79	5.04	2.82	4.16	2.85
H19	2.21	1.47	5.16	2.94	5.11	3.03	4.28	2.67	4.89	2.69
H29	3.79	2.46	5.00	2.71	4.32	2.52	4.96	2.75	4.63	2.69
H35	2.42	2.06	5.40	2.58	5.16	2.89	5.72	2.62	4.79	2.39
Trick1	2.26	1.97	4.36	2.33	4.11	2.83	5.08	2.43	4.47	2.80
Trick11	2.16	1.57	5.32	2.79	4.47	2.89	5.52	2.77	4.95	2.70
Trick25	2.63	1.86	4.29	2.79	3.95	2.44	4.88	2.80	4.11	2.31
Trick33	1.79	1.23	5.33	2.63	4.63	2.77	5.75	3.04	4.84	2.83
Trick41	2.21	1.93	3.54	2.78	3.47	2.78	3.71	2.85	3.32	2.79

We first computed the means of each video clip separately for the two groups and then computed the descriptive statistics. It has been reported the number of participants for each group that evaluated each video clip and mean and standard deviation for all variables.
